# FLASH instead of proton arc therapy is a more promising advancement for the next generation proton radiotherapy

**DOI:** 10.1002/acm2.14091

**Published:** 2023-07-11

**Authors:** Minglei Kang, Xuanfeng Ding, Yi Rong

**Affiliations:** ^1^ New York Proton Center New York New York USA; ^2^ Department of Radiation Oncology Corewell Health William Beaumont University Hospital Royal Oak Michigan USA; ^3^ Department of Radiation Oncology Mayo Clinic Phoenix Arizona USA

**Keywords:** FLASH, Proton arc therapy

## 1 INTRODUCTION

In the clinical practice of radiation oncology, we rely on precise dose calculation and deposition,^1^, ^2^, ^3^ high dose conformity^4^, ^5^, ^6^, ^7^ to the target while lowering the dose to the adjacent Organs‐At‐Risk (OARs) as much as possible. With the improvement in technology development and high computation power for particle radiotherapy, researchers are focusing mainly on two aspects, increasing the degree of freedom in plan optimization for an arc delivery^8^ and increasing the dose rate to reach the so‐called “FLASH” level.^9^ While both techniques may be able to drive the proton RT field to a more advanced level, the question that comes to our mind is which one is more promising in becoming the mainstream of the next generation proton treatment. Herein, we invited two experts in the field to provide their opinions. We have Dr. Minglei Kang arguing for the proposition that “FLASH instead of proton Arc Therapy is a more promising advancement for the next generation proton radiotherapy,” while Dr. Xuanfeng Ding arguing against.

Dr. Kang is the Lead Medical Physicist and associate research professor at the New York Proton Center (NYPC). He obtained his PhD in accelerator physics with distinction from Peking University. After doctoral graduation in 2011, he was appointed as a medical physicist and research assistant professor at the Chinese Academy of Medical Sciences Cancer Hospital. He then came to the University of Pennsylvania, where he completed a postdoctoral fellowship and medical physics residency training. Before joining NYPC in 2018, Dr. Kang served as an assistant professor and medical physicist at Georgetown University. He actively contributes to several committees and consortia, including the American Associate of Physicists in Medicine (AAPM) task group (TG) 349, Journal of Applied Clinical Medical Physics, Particle Therapy Cooperative Group (PTCOG) Thoracic and Gastrointestinal Subcommittee, and NRG Oncology Liver Proton SBRT Working Group. His research focuses on proton system commissioning, Monte Carlo, planning optimization, motion management, small‐field dosimetry, FLASH therapy, and so on.

Dr. Ding received his PhD in Physics from Wake Forest University in 2012 and finished his residency training at the University of Pennsylvania in 2014. Dr. Ding is the lead proton physicist and associate professor at Corewell Health, William Beaumont University Hospital, Royal Oak. His research interests include the proton arc technique, adaptive therapy, and motion management. He received several extramural research grants as the PI and was granted multiple patents. Dr. Ding published over 40 peer‐reviewed papers and hundreds of conference abstracts. He is certified by the American Board of Radiology in Therapeutic Radiologic Physics. He was co‐chair of the European Society of Radiotherapy and Oncology (ESTRO) physics workshop: Particle Arc Therapy in 2022, president of the Great Lakes Chapter AAPM in 2020, and committee member of several AAPM task and work groups.

## 2 OPENING STATEMENTS

Minglei Kang, PhD

Proton radiation therapy (RT) is a promising treatment modality for cancer patients, providing highly targeted tumor treatment while minimizing damage to healthy tissue by utilizing the Bragg peaks of proton beams.^10^, ^11^ Two innovative approaches, proton FLASH RT and proton arc RT, have emerged.^8^, ^9^ While both methods hold promise, I believe proton FLASH RT is the superior option.

Proton FLASH RT is an innovative approach that delivers therapeutic doses at an ultrahigh dose rate exceeding 40 Gy/s.^9^ This technique has garnered significant attention due to its promising outcomes in sparing normal tissue while maintaining comparable effectiveness in killing cancer cells. Numerous studies have demonstrated the preservation of functionality in various anatomical sites, such as the lung,^9^ skin,^12^ brain,^13^, ^14^, ^15^ and abdomen.^16^ The initial human study involving a CD30+ T‐cell cutaneous lymphoma patient treated with FLASH beams showed promising results in terms of protecting normal skin and eliciting a positive tumor response.^17^ The first clinical trial of proton FLASH RT was conducted on a group of 10 patients who were experiencing symptomatic bone metastases.^18^ The trial successfully assessed the effectiveness of pain relief, treatment workflow, and safety using proton pencil beam scanning (PBS) FLASH RT. This body of evidence suggests that FLASH RT can provide substantial benefits over conventional RT methods, including an improved therapeutic window, reduced side effects, efficient delivery, and shortened treatment duration. More specifically, it represents a revolutionary approach with promising outcomes in preclinical studies, suggesting its potential to provide substantial clinical benefits.^19^ Secondly, it holds the capability to considerably reduce treatment durations, which can be particularly advantageous for patients with aggressive cancers. Thirdly, it can potentially mitigate the risk of radiation‐induced side effects, such as damage to healthy tissues and organs.

On the contrary, proton arc RT employs rotating sub‐beams to deliver prescribed doses in a continuous arc, enabling enhanced conformity and precision. Although the feasibility of arc RT has been demonstrated on a clinical proton system, it remains in the experimental stages.^8^ Unlike FLASH RT, arc RT does not deviate from the fundamental principles of radiation therapy for tissue protection; instead, it employs a rotational beam approach rather than fixed beams from multiple angles. However, concerns persist regarding its effectiveness, efficiency, and safety. Furthermore, proton arc RT delivers the prescribed doses at a conventional dose rate, indicating that its effectiveness for cancer treatment is expected to be comparable to the current standard of care in proton therapy. While it does offer certain advantages over traditional delivery methods, the benefits it provides for cancer treatment are relatively limited. The standard of care in PBS proton therapy^20^, ^21^, ^22^ and the single‐energy Bragg peak FLASH technique^23^, ^24^, ^25^, ^26^ already enable the delivery of highly conformal and precise radiation doses to tumors, which diminishes the significance of proton arc RT for future applications.

In conclusion, proton FLASH RT represents a significant advancement in the field of proton radiation therapy and holds great promise for cancer treatment. While proton arc RT may offer dosimetric advantages for special treatment scenarios, it may not bring fundamental changes to the current or future practice as promised by the proton FLASH. Its safety and efficiency need to be justified. For these reasons, I believe that proton FLASH RT is a more promising option for the next generation approach for cancer patients.

Xuanfeng Ding, PhD

FLASH is an exciting treatment modality that has the potential to bring a revolutionary radiobiology effect into the current clinical RT practice. However, if we look back at the last half‐century, our radiation oncology communities benefited more directly from the engineering and technological evolution and increased degree of freedom, from 2D technique, 3D conformal therapy,^27^ Intensity Modulated Radiotherapy (IMRT),^28^, ^29^ Volumetric Modulated Arc Therapy (VMAT),^30^, ^31^, ^32^ ultimately to the 4pi approach.^33^, ^34^, ^35^ It does not mean that the radiobiology effect is not important, but the reality is that engineering and technological development improves the dose delivery accuracy, treatment efficiency and target conformity, which are noticeably associated with significant improvements in the quality of life for cancer patients, such as toxicity reductions and better tumor control probability.^36^, ^37^, ^38^, ^39^, ^40^ Thus, these advanced techniques have been quickly adopted as a clinical routine and dominate the market without needing to re‐invent the wheels in the radiobiology model.^41^, ^42^, ^43^ Frankly speaking, we knew the importance of the degree of freedom from the beginning of the journey in radiotherapy when Dr. Leksell invented Gamma Knife, where hundreds of cobalt 60 sources focused the gamma‐ray on the tiny targets.^33^ Nowadays, VMAT utilizing the arc trajectories is dominating the routine practice via C‐arm typed LINACs,^35^, ^44^ and 4pi approaches are dominating in the cranial region where a superior dose fall‐off is preferred, even though the radiobiology is still not fully understood in the hypofractionation regimens.^5^, ^45^, ^46^

Unfortunately, the importance of an increased degree of freedom has been somewhat overlooked in particle beam therapy's routine clinical practice, where the fantasy about utilizing a couple of beam angles to spare the healthy tissue via the Bragg peak has been dominating the community in the last half‐century,^47^, ^48^, ^49^, ^50^ One of the major concerns of utilizing more beam angles is the spilling of the low dose to the healthy tissue, which has not been seen or studied before, and the excessive low dose volume may defeat the purpose of using particle beam therapy.^38^, ^51^, ^52^ Another major concern is that the current technology might not be able to support the massive dynamic rotational gantry while delivering the spot within a submillimeter accuracy. Thus, an experimental rotational platform was designed and tested in combination with a fixed beam line.^53^ Additionally, it seems infeasible to directly generate a robust arc plan with hundreds of control points due to the heavy calculation burden, as it already takes tremendous time to optimize a robust Intensity Modulated Proton Therapy (IMPT) plan with a couple of fields in early 2010s.^8^ As a result, the concept of particle arc therapy was not at the center of the research development until the introduction of the first robust spot‐scanning particle arc therapy optimization algorithm^8^ and the demonstration of its feasibility using a clinical system.^54^

Based on this concept, publications from different research institutions have shown the potential clinical benefit of proton arc therapy for a wide range of clinical indications,^55^, ^56^, ^57^, ^58^, ^59^, ^60^, ^61^, ^62^ drawing significant interest among radiation oncology communities, including the ESTRO,^63^ AAPM,^64^ and PTCOG.^65^ Multiple international consortiums have been established, aiming to join‐force in pushing the technique forward.^66^, ^67^ The proton system manufacturers such as IBA are taking the key steps to such development, while the Treatment Planning System (TPS) providers such as RaySearch and Elekta are also providing the proton arc module in support of future clinical implementations.^68^, ^69^ Additionally, many ideas have been formed to optimize particle arc therapy in a more efficient way.^70^, ^71^, ^72^, ^73^, ^74^, ^75^ In recent years, we saw exponentially increased publications on the topic of particle arc therapy. These encouraging trends and global efforts might suggest that the proton RT is at an important turning point, similar to the invention of VMAT in the 2000s in the photon world.^30^

Besides the potential clinical benefits, one driving factor that pushes particle arc therapy to dominate the future market is simplifying the clinical workflow and shortening the treatment delivery time.^76^ This key feature will enable the proton therapy center to treat more patients, which is critical in today's challenging financial situation, especially with a huge investment and high operation cost.^77^, ^78^ In other words, more cancer patients will be benefited from arc therapy with such precious medical resources. A similar phenomenon was observed in the adoption of VMAT, even though there is no clear level 1 evidence of superior clinical benefits compared to the IMRT.^79^ The effectiveness of the Linear Energy Transfer (LET) optimization via the arc trajectory not only spares the critical OARs from the high LET region but also increases the possibility of LET escalation in the tumor center where the radiation‐resistant tumor cells (hypoxic) are usually located.^80^ This feature would be critical for carbon arc therapy, where the RBE calculation is based on the LET.^81^ While pushing particle beam therapy toward future biological optimization, the arc therapy platform paves the foundation, flexibility, and feasibility for such exploratory investigation. Admittedly, it is still a long way to go, but it would be a beautiful and exciting journey toward rotational arc therapy in the following decades.

In summary, the clinical adoption of the rotation arc therapy technique will be a natural step in improving the dosimetric plan quality, treatment efficiency, and biological optimization with the ultimate goal of improving the quality of life for cancer patients. In the next ten years, we will see particle arc treatments ramping up at the new particle therapy centers and upgrade requests from the existing centers. On the other hand, FLASH has been a promising research direction that will lead us to an entirely new and revolutionary biology world. However, it may take decades before we can systematically understand the principles behind the FLASH phenomenon. Before that, a wide clinical adoption or routine clinical implementation of proton FLASH therapy in the next decades seems impossible.

## 3 REBUTTAL

Minglei Kang, PhD

While acknowledging the benefit from both proton arc therapy^8^ and proton FLASH,^82^, ^83^ this rebuttal aims to provide a more balanced perspective by addressing certain points and highlighting the potential of proton FLASH for wider clinical implementation that will be the dominating technique for proton radiotherapy.

While proton arc therapy certainly offers advantages, such as enhanced dosimetric conformity and expanded patient eligibility,^84^ it is crucial to recognize that proton FLASH therapy also has the potential to revolutionize the field. The ultra‐high dose‐rate delivery of proton FLASH therapy enables shorter treatment times, reduced toxicities, and improved patient comfort, presenting compelling advantages that should not be overlooked. My opponent's claim suggests that proton arc therapy's adherence to fundamental principles and incremental adoption based on existing technology is an advantage. While it is true that familiarity can facilitate adoption, it may also limit the potential for groundbreaking advancements. Given that the present PBS IMPT already demonstrates superior dosimetric outcomes compared to photon and proton scattering techniques,^10^, ^85^ there is limited urgency to invest significant efforts into the development of proton arc therapy. Proton FLASH therapy, on the other hand, introduces a paradigm shift by challenging conventional assumptions and exploring the unique benefits of ultra‐high dose‐rate radiation. Embracing such transformative approaches can lead to significant leaps in treatment efficacy and patient outcomes.

It is important to recognize that proton FLASH therapy has demonstrated its effectiveness and efficiency in preclinical studies, showing equivalent tumor control while minimizing damage to healthy tissues.^9^, ^18^ The ongoing research and clinical trials in the field of proton FLASH therapy are actively addressing the remaining questions and paving the way for its clinical implementation.^19^, ^83^ While certain in silico simulations suggest potential dosimetric benefits of proton arc therapy,^55^, ^56^, ^57^, ^58^, ^77^, ^86^ there is currently no evidence demonstrating its translation into biological advantages. The improved conformity achieved through arc therapy comes at the expense of sacrificing the advantages of Bragg peaks offered by proton beams, resulting in increased radiation doses to healthy tissues. In my opinion, the current IMPT technique can effectively optimize all clinical cases, including the most challenging ones. In essence, arc therapy may offer a similar solution, but not necessarily a more effective one. Therefore, even if the safety of arc therapy can be justified through future technical advancements, its anticipated effectiveness and efficiency remain relatively insignificant. Proton FLASH therapy has shown promising results, and ongoing investigations aim to address the remaining challenges.^18^, ^19^, ^83^ With substantial resources and time devoted to refining the FLASH irradiation system, quality assurance devices, and radiation biology models, the path toward clinical implementation is being actively pursued.^87^, ^88^, ^89^, ^90^

Proton FLASH therapy may gain momentum as more clinical evidence and biological data are accumulated. These two developments are not mutually exclusive, and their coexistence can bring a new era of fast, efficient, precise, and effective personalized therapy. In summary, while proton arc therapy offers familiarity and incremental innovation, proton FLASH therapy presents unique advantages that may revolutionize the field. Continued research, investment, and clinical trials in proton FLASH therapy will contribute to a comprehensive understanding of its effectiveness, efficiency, and safety, ultimately shaping the future of advanced radiation therapy. Therefore, proton FLASH exhibits the greatest potential to emerge as the prevailing technique for the next generation of advanced proton radiotherapy treatment.

Xuanfeng Ding, PhD

I fully agree with my opponent that *“proton arc therapy does not deviate from the fundamental principles of the radiation therapy,”* and the beauty of proton arc therapy is that our radiation oncology community could quickly adopt it for clinical use. As such an innovation is built upon the pencil beam scanning technology widely used in clinical settings, we can move one step at a time before jumping into the unknown FLASH effectiveness world. On the other hand, before any new technology is clinically implemented, we must address these questions *“effectiveness, efficiency, and safety.”* With the mounting global interests, multi‐institutional efforts, and investigations, the merging data shine a light on the effectiveness and efficiency improvements where a 30% increase in the daily patient treatment throughput is now expected for a single room system.^76^ Based on the Normal Tissue Complication Probability (NTCP) model prediction, an extra 15% of oropharyngeal cancer patients in the Netherland will be qualified and benefit from using proton arc therapy.^84^ In addition, numerous disease sites and clinical indications could potentially benefit from proton arc therapy through *in silico* simulations.^55^, ^56^, ^57^, ^58^, ^60^, ^61^, ^86^ As we move closer to the clinical implementation of proton arc therapy, our vendors, including proton therapy machines, treatment planning systems, and quality assurance devices, are motivated to develop the best product solution for a safe and effective clinical operation. I am optimistic that these questions will be fully answered in the next couple of years.

FLASH therapy, on the other hand, does allow for establishing a revolutionary approach in radiotherapy.^91^ But we also need to acknowledge that it is a high‐risk and high‐reward direction in which enormous resources and time must be spent to develop a robust and reliable FLASH irradiation system, QA devices, and re‐establish radiation biology effectiveness model incorporating complicated FLASH effects.^92^ Today we are still in the preliminary stage of testing the hypothesis, such as oxygen depletion,^93^ immune and inflammatory processes,^94^ or combined.^82^ For the next 10−30 years, I am not optimistic about the implementation of FLASH therapy for a wide range of clinical indications as proton arc therapy would do. However, it is important for our government agency to continue to invest in and support such research directions and clinical trials, allowing our scientific community to untangle the puzzles for the cancer population's long‐term benefits.^83^

Things may change, or maybe I might be wrong that in FLASH, we do not need to be too conservative or dig into the precise modeling of the FLASH biological effect. Maybe FLASH could be as successful as SBRT in which, even after two decades of clinical adoption, we still do not fully understand the radiobiology in the hypofractionation regimen, and the clinical outcome is excellent.^95^ However, I think until then, proton arc therapy will be ready to be compatible with FLASH offering a SPLASH technique.^96^

In summary, Proton arc therapy is poised to emerge as the most advanced technique for next‐generation treatment. FLASH may be followed as we accumulate more clinical evidence and experimental biological data along the roadmap. The good part is that both developments will push our radiation treatment technology to its limits toward a new era of fast, efficient, precise, and effective personalized therapy.

## AUTHOR CONTRIBUTION

XD, MK and YR initiate the topic and discussion.

## CONFLICT OF INTEREST STATEMENT

Dr. Ding received industry research funding from IBA, Elekta, and RadioMed outside the work presented here. Dr. Ding holds a patent related to proton arc therapy which has been licensed to IBA. Dr. Kang received a Varian research grant outside of this work.
